# Human Skin-Derived Mast Cells Spontaneously Secrete Several Angiogenesis-Related Factors

**DOI:** 10.3389/fimmu.2019.01445

**Published:** 2019-06-25

**Authors:** Cody McHale, Zahraa Mohammed, Gregorio Gomez

**Affiliations:** Department of Pathology, Microbiology and Immunology, University of South Carolina School of Medicine, Columbia, SC, United States

**Keywords:** mast cells, angiogenesis, tumorigenesis, VEGF, stem cell factor, FcεRI

## Abstract

Mast cells are classically recognized as cells that cause IgE-mediated allergic reactions. However, their ability to store and secrete vascular endothelial growth factor (VEGF) suggests a role in vascular development and tumorigenesis. The current study sought to determine if other angiogenesis-related factors, in addition to VEGF, were also secreted by human tissue-derived mast cells. Using proteome array analysis and ELISA, we found that human skin-derived mast cells spontaneously secrete CXCL16, DPPIV, Endothelin-1, GM-CSF, IL-8, MCP-1, Pentraxin 3, Serpin E1, Serpin F1, TIMP-1, Thrombospondin-1, and uPA. We identified three groups based on their dependency for stem cell factor (SCF), which is required for mast cell survival: Endothelin-1, GM-CSF, IL-8, MCP-1, and VEGF (dependent); Pentraxin 3, Serpin E1, Serpin F1, TIMP-1, and Thrombospondin-1 (partly dependent); and CXCL16, DPPIV, and uPA (independent). Crosslinking of FcεRI with multivalent antigen enhanced the secretion of GM-CSF, Serpin E1, IL-8, and VEGF, and induced Amphiregulin and MMP-8 expression. Interestingly, FcεRI signals inhibited the spontaneous secretion of CXCL16, Endothelin-1, Serpin F1, Thrombospondin-1, MCP-1 and Pentraxin-3. Furthermore, IL-6, which we previously showed could induce VEGF, significantly enhanced MCP-1 secretion. Overall, this study identified several angiogenesis-related proteins that, in addition to VEGF, are spontaneously secreted at high concentrations from human skin-derived mast cells. These findings provide further evidence supporting an intrinsic role for mast cells in blood vessel formation.

## Introduction

Mast cells are hematopoietic tissue resident immune cells that are classically recognized as the main effector cell type of Immunoglobulin E (IgE)-mediated immediate hypersensitivity reactions (1, 2). In addition to their classical role in allergy, mast cells are frequently associated with tumors in humans, and are implicated in tumorigenesis (3). The exact role of mast cells in tumor formation is not known. However, tumor-associated mast cells (TAMCs) have been shown to have both pro- and anti-tumorigenic effects in human tumors (3), and it appears that the overall impact of mast cells on tumor formation is tissue dependent (4). For example, mast cells have pro-tumor effects in human cancers of the thyroid (5, 6), stomach (7–9), and bladder (10). In contrast, mast cells appear to protect against breast cancer (11–13). In most cases, however, both pro- and anti-tumorigenic effects of mast cells have been reported, such as in cancers of lung, colon, pancreas, and prostate. Thus, mast cells appear to have both positive and negative effects on tumor formation.

Mast cells are a major source of cytokines, chemokines, and growth factors that can contribute to tumor development (14, 15). It has been known for some time that mast cells contain VEGF within their cytoplasmic granules, and can secrete this growth factor both spontaneously and following activation (16, 17). These early observations were made in murine bone marrow-derived mast cells (BMMCs), human cord blood-derived mast cells (CBMCs), mast cells from human foreskin, and the HMC-1 cell line. In addition, we recently demonstrated the spontaneous, IL-6- and FcεRI-induced release of VEGF from *in situ*-matured mast cells from human breast and abdominal skin (18). Several studies have also shown the expression of VEGF transcripts in various primary mast cells and cell lines from human and mouse (19–21). In each case, primary mast cells were shown to express transcripts for pro-angiogenic VEGF-A and VEGF-B, and pro-lymphangiogenic VEGF-C and VEGF-D. In addition to FcεRI signals, IL-6 (18), IL-9 (22), PGE_2_ (20), Cordicotropin Releasing Hormone (23), and adenosine (24) can also induce VEGF secretion from mast cells. Mast cell-derived VEGF was shown to induce proliferation and migration of human endothelial cells (16), and to induce angiogenesis in the chick embryo chorioallantoic membrane assay (19). Other factors, including Angiopoietin-1 (21), have also been implicated in mast cell-mediated angiogenesis and tumor growth (3, 15, 25). Nevertheless, most studies have focused on VEGF as the primary promoter of tumorigenesis from mast cells.

Here, we demonstrate that tissue-derived mast cells from human skin spontaneously secrete several angiogenesis-related factors, in addition to VEGF, at high concentrations. Specifically, we identified CXCL16, DPPIV, Endothelin-1, GM-CSF, IL-8, MCP-1, Pentraxin 3, Serpin E1, Serpin F1, TIMP-1, Thrombospondin-1, and uPA as being spontaneously secreted at levels greater than VEGF. Secretion of some factors required stem cell factor (SCF) whereas others were secreted completely independent of the survival factor. We show that FcεRI crosslinking enhances, but also inhibits, the secretion of several factors. Lastly, we show that IL-6 induced the secretion of MCP-1. Together, these data demonstrate that mast cells are a major source of several different angiogenesis-regulating factors. Thus, supporting the notion of an intrinsic role for mast cells as regulators of blood vessel formation (15).

## Materials and Methods

### Isolation, Purification, and Culture of Human Skin Mast Cells

Mast cells were isolated and purified from fresh surgical specimens of human skin tissues that were purchased from the Cooperative Human Tissue Network (CHTN) of the National Cancer Institute. These studies were approved by the human studies Internal Review Board (IRB) of University of South Carolina. The tissues, which were received within 24 h post-surgery, were mechanically minced with surgical scissors, and then digested with collagenase type II, hyaluronidase, and DNase I in HBSS buffer (1X HBSS, 0.04% NaHCO_3_, 1% fetal bovine serum, 1% HEPES, 0.1% CaCl_2_) containing Amphotericin B and Antibiotic/Antimycotic solution. A total of 3 × 1 h digestions at 37°C were performed. The samples were filtered through 40 μm nylon cell strainers after each digestion, and the dispersed cells were collected by centrifugation. The cells were separated on a Percoll cushion by density centrifugation. The cells at the interface of buffer and Percoll layers were collected, washed and re-suspended at 5 × 10^5^ cells/ml in serum-free X-VIVO 15™ media (Lonza) supplemented only with recombinant human stem cell factor (SCF, 100 ng/ml) (PeproTech). A small aliquot is usually stained with Toluidine Blue in order to get a rough idea of the starting number of mast cells obtained, which is ~5–7% of the total population based on our extensive experience. Total cells were transferred onto 24-well plates and maintained under standard culture conditions (37°C, 5% CO_2_) with weekly media changes and transfer to new plates as necessary. At ~4–6 weeks of culture, most of the non-mast cells have died-off leaving a majority of mast cells. Purity was assessed by metachromatic staining with acidic toluidine blue, and by immunofluorescence staining for FcεRI expression with PE-labeled anti-human FcεRI antibody [clone AER-37 (CRA)] and mouse IgG2b_k_ isotype control (BioLegend). The mast cells were used only when >95% of the cells were FcεRI^+^ (~8 weeks of culture). A published diagram of the isolation procedure with representative figures can be found in Troupin et al. (26).

### Proteome Arrays

Cell-free supernatants were profiled with the membrane-based Human Angiogenesis Proteome Profiler™ Array (R&D Systems) according to the manufacturers' instructions. The blots were scanned on an Odyssey® CLx Infrared Imaging System (LI-COR Biosciences).

### Cytokine Measurements

Cytokines in cell-free medium were measured with enzyme-linked immunosorbent assay (ELISA) kits (R&D Systems) according to the manufacturers' instructions. Absorbance values were obtained with a BioTek Synergy HT microplate reader, and cytokine concentrations were determined using Gen5 Data Analysis Software.

### IgE Sensitization and FcεRI Crosslinking

Mast cells were incubated in X-VIVO 15™ media containing SCF (100 ng/ml) and chimeric human IgE anti-NP (1 μg/10^6^ cells) (clone JW8/1; AbD Serotec) overnight at 37°C, 5% CO_2_. The cells were washed and re-suspended at 10^6^ cells/ml in X-VIVO 15™ media and activated with the hapten 4-hydroxy-3-nitrophenylacetyl conjugated to bovine serum albumin at a 16:1 molar ratio (NP-BSA; Biosearch Technologies) at the indicated concentration at 37°C for the indicated amount of time.

### Statistical Analysis

Statistical analysis was performed using GraphPad Prism version 6.0c for Mac OS X, GraphPad Software, La Jolla California USA, www.graphpad.com.

## Results

### Human Skin-Derived Mast Cells Constitutively Secrete Several Angiogenesis-Related Factors

**Figure 1:**
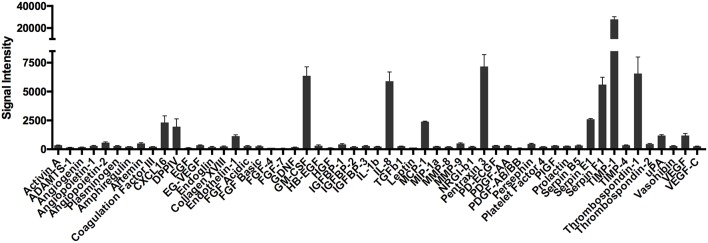
Proteome array analysis of spontaneously secreted angiogenesis-related proteins from human skin mast cells. Human skin mast cells were cultured for 24 h in serum-free media containing only SCF and SBTI, and the cell-free supernatants were analyzed with the Human Angiogenesis Proteome Profiler™ Array (R&D Systems). The graph bars represent the mean signal intensity ± S.E.M. (*n* = 4) obtained from membrane arrays incubated with media from individual mast cell cultures prepared from skin tissue of different donors.

It is well-established that mast cells store and spontaneously secrete VEGF (16, 17). In our studies, VEGF was consistently detected at high levels in supernatants from non-activated human skin mast cells (18). Therefore, we sought to determine if other angiogenesis-related proteins, in addition to VEGF, were also secreted constitutively from tissue-derived mast cells. To do so, human skin-derived mast cells were washed and cultured in serum-free medium containing only stem cell factor (SCF), which is required for differentiation and survival of mast cells (27–31), and soybean trypsin inhibitor (SBTI) to prevent proteolysis of secreted proteins by endogenous proteases (32). After a 24 h incubation period, the cell-free medium was analyzed with the Human Angiogenesis Proteome Profiler™ Array (R&D Systems), which enabled us to detect 55 different angiogenesis-related proteins. To our surprise, several proteins—CXCL16, DPPIV, Endothelin-1, GM-CSF, IL-8, MCP-1, Pentraxin 3, Serpin E1, Serpin F1, TIMP-1, Thrombospondin-1, and uPA—were detected at surprisingly high levels with this proteome array analysis (Figure 1). It was particularly interesting that these proteins were secreted at levels much greater than VEGF.

**Figure 2:**
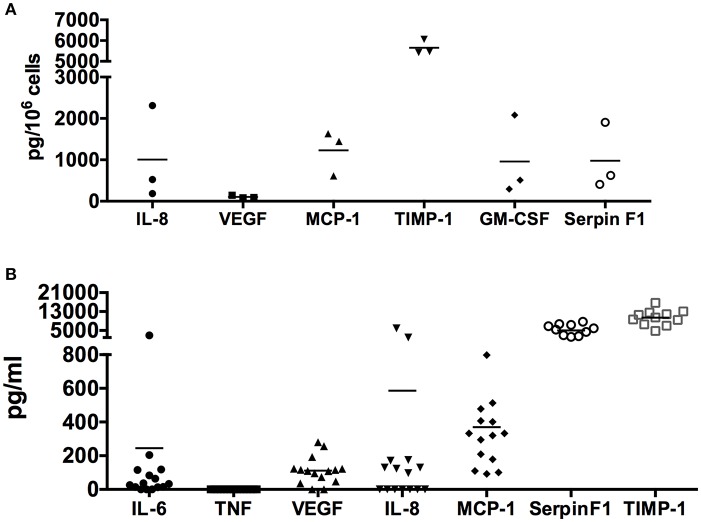
Quantification of spontaneously secreted angiogenesis-related proteins from human skin mast cells. Human skin mast cells prepared from individual donor tissues were cultured in serum-free media for 24 h **(A)** (*n* = 3 donor tissues) or 7 days **(B)** (*n* = 11–15 donor tissues), and the cell-free supernatants were analyzed for IL-8, VEGF, MCP-1, TIMP-1, GM-CSF, and Serpin F1 with ELISA. IL-6 and TNF were analyzed as positive and negative controls, respectively. The 24-h culture media **(A)** contained SCF + SBTI whereas the 7-days culture media **(B)** contained only SCF. These data verify the proteome array analysis, and quantify the amount of protein spontaneously secreted.

To validate the proteome profiler array data, we cultured human skin-derived mast cells from different donor tissues in serum-free medium containing only SCF and SBTI for 24 h, and measured IL-8, VEGF, MCP-1, GM-CSF, TIMP-1, and Serpin F1 with ELISA. As shown in Figure 2A, all proteins analyzed were detected at quantifiable levels after 24 h in culture under non-stimulated conditions. Importantly, TIMP-1 and VEGF, which were detected by proteome array at high and low levels, respectively, were also detected at high and low quantities with ELISA. Thus, the ELISA data essentially mirrors the relative signal intensities of the proteome array.

In addition, we also determined the concentration of IL-8, VEGF, MCP-1, TIMP-1, and Serpin F1 in media from cultures of resting skin-derived mast cells collected during routine (every 7 days) media changes. IL-6 and TNF were also analyzed as positive and negative controls, respectively, since previous studies had shown that IL-6 but not TNF was spontaneously secreted by human skin mast cells (18, 33). As shown in Figure 2B, IL-8, VEGF, MCP-1, GM-CSF, TIMP-1, and Serpin F1 were all detected in the cell free medium. In agreement with the proteome array data, TIMP-1 and Serpin F1 were detected at extremely high concentrations, followed by MCP-1, IL-8, and VEGF. As expected, IL-6 (positive control) was detected whereas TNF (negative control) was not. It is worth noting that the time in culture, and cell densities of the different mast cell cultures was variable at the time the media was collected, and that SBTI, which is not usually added to the culture media, was not present in the media collected from the established cultures. Together, these findings demonstrate that human skin-derived mast cells spontaneously secrete a variety of angiogenesis-related proteins, in addition to VEGF, at high levels in the absence of any exogenously added stimuli.

### Dependence on Stem Cell Factor

**Figure 3:**
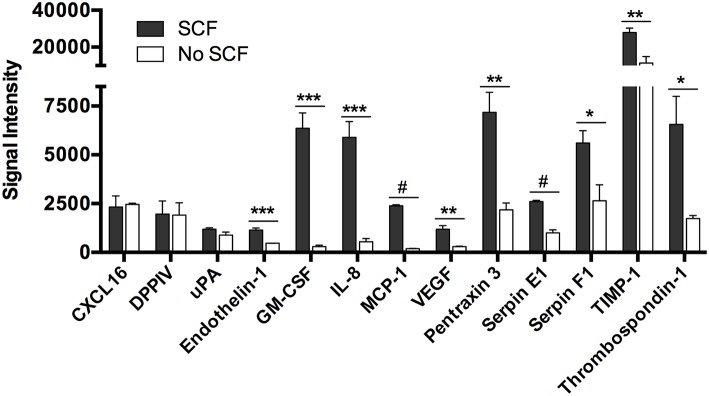
Spontaneous secretion of angiogenesis-related proteins from human mast cells is variably dependent on stem cell factor. Human skin mast cells were cultured in serum-free media containing SBTI ± SCF for 24 h, and the cell-free supernatant was analyzed with the Human Angiogenesis Proteome Profiler™ Array (R&D Systems). The graph bars represent the mean signal intensity ± S.E.M. (*n* = 4) obtained from membrane arrays incubated with media from individual mast cell cultures prepared from skin tissue of different donors. Significance was determined with Student's *t*-test. ^*^p < 0.05; ^**^p < 0.01; ^***^p < 0.01; ^#^*p* < 0.001.

To determine if secretion of the angiogenesis-related factors was due to stimulation of c-kit by exogenously added SCF, we cultured human skin-derived mast cells with and without SCF (100 ng/ml) in serum-free media containing only SBTI for 24 h, and analyzed the cell-free medium with the Human Angiogenesis Proteome Profiler™ Array. As shown in Figure 3, there was no difference in secretion of CXCL16, DPPIV, and uPA in the presence or absence of SCF, whereas endothelin-1, GM-CSF, IL-8, MCP-1, and VEGF secretion was almost completely abolished in the absence of SCF. In addition, secretion of Pentraxin 3, Serpin E1, Serpin F1, TIMP-1, and Thrombospondin-1 was significantly reduced, but still detected at very high levels in the absence of SCF. Thus, we have identified three groups of angiogenesis-related proteins whose secretion is independent (CXCL16, DPPIV, and uPA), dependent (endothelin-1, GM-CSF, IL-8, MCP-1, and VEGF), or somewhat dependent (Pentraxin 3, Serpin E1, Serpin F1, TIMP-1, and Thrombospondin-1) on SCF.

### FcεRI Crosslinking Augments and Inhibits Secretion of Angiogenesis-Related Proteins

**Figure 4:**
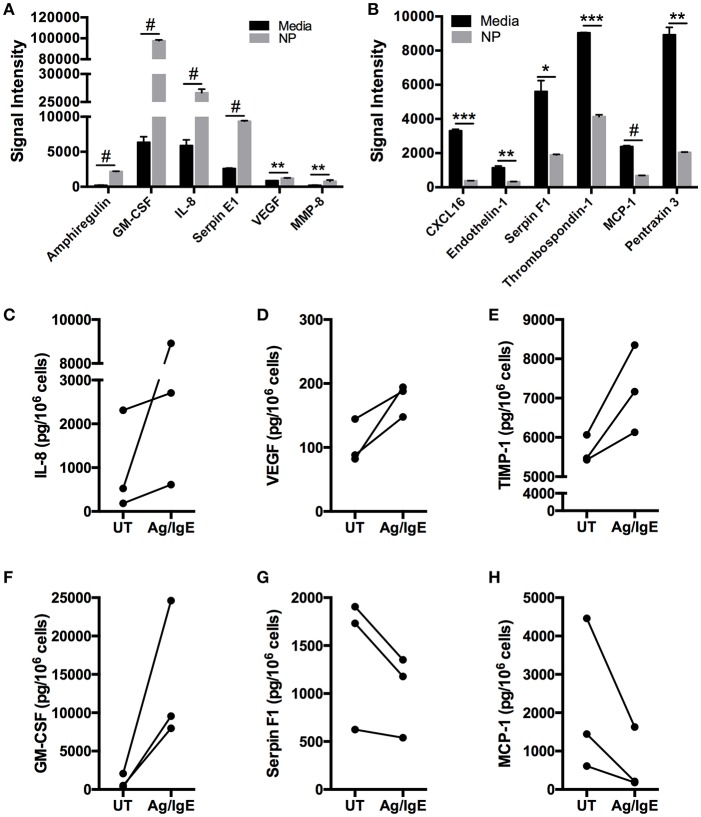
FcεRI crosslinking enhances and inhibits human mast cell secretion of angiogenesis-related factors. Human skin mast cells were sensitized with anti-DNP IgE and then activated with DNP-HSA (100 ng/ml) for 24 h in serum-free media containing SCF and SBTI. The cell-free supernatants were analyzed with the Human Angiogenesis Proteome Profiler™ Array (R&D Systems) **(A,B)**. The graph bars represent the mean signal intensity ± S.E.M. (*n* = 4) obtained from membrane arrays incubated with media from individual mast cell cultures prepared from skin tissue of different donors. In separate experiments with different mast cell cultures, media was collected and analyzed with ELISA for IL-8 **(C)**, VEGF **(D)**, TIMP-1 **(E)**, GM-CSF **(F)**, Serpin F1 **(G)**, and MCP-1 **(H)**. The data points in **(C–H)** represent values obtained from mast cells isolated from individual skin tissue of three different donors. Significance was determined with Student's *t*-test. ^*^p < 0.05; ^**^p < 0.01; ^***^p < 0.01; ^#^*p* < 0.001.

To determine the effect of FcεRI crosslinking on secretion of angiogenesis-related proteins particularly those found to be spontaneously secreted, human skin-derived mast cells were sensitized with anti-DNP IgE, and then challenged with DNP-HSA (100 ng/ml) for 24 h. The cell-free medium was collected, and analyzed with the Human Angiogenesis Proteome Profiler™ Array as in the previous experiments. As shown in Figure 4A, FcεRI crosslinking increased the secretion of GM-CSF, IL-8, Serpin E1, and VEGF, and induced the secretion of Amphiregulin and MMP-8. Surprisingly, FcεRI crosslinking also resulted in significant reduction in spontaneous secretion of CXCL16, Endothelin-1, Serpin F1, Thrombospondin-1, MCP-1, and Pentraxin-3 (Figure 4B). To confirm the proteome array data, the experiment was repeated and IL-8, VEGF, TIMP-1, GM-CSF, Serpin F1, and MCP-1 in the cell-free supernatants were quantified with ELISA (Figures 4C–H). Confirming the proteome array data, IL-8, VEGF, TIMP-1, and GM-CSF were increased following FcεRI crosslinking, whereas Serpin F1 and MCP-1 secretion was inhibited.

It is well-known that FcεRI signals upregulate cytokine gene expression and secretion. Therefore, it was surprising that the spontaneous secretion of some proteins was inhibited following FcεRI crosslinking. To rule out the possibility that free NP-BSA in the sample interfered with the antigen-antibody binding in the ELISA assay, we generated standard curves of MCP-1 and Serpin F1 in the presence and absence of NP-BSA (100 ng/ml). We observed no significant shift in the curves with NP-BSA (Figure S1), indicating that the observed FcεRI-induced inhibition in secretion was not likely due to interference by free NP-BSA with the ELISA. Thus, FcεRI crosslinking augments and inhibits the spontaneous secretion of angiogenesis-related factors from mast cells.

### IL-6 Induces MCP-1 Secretion

**Figure 5:**
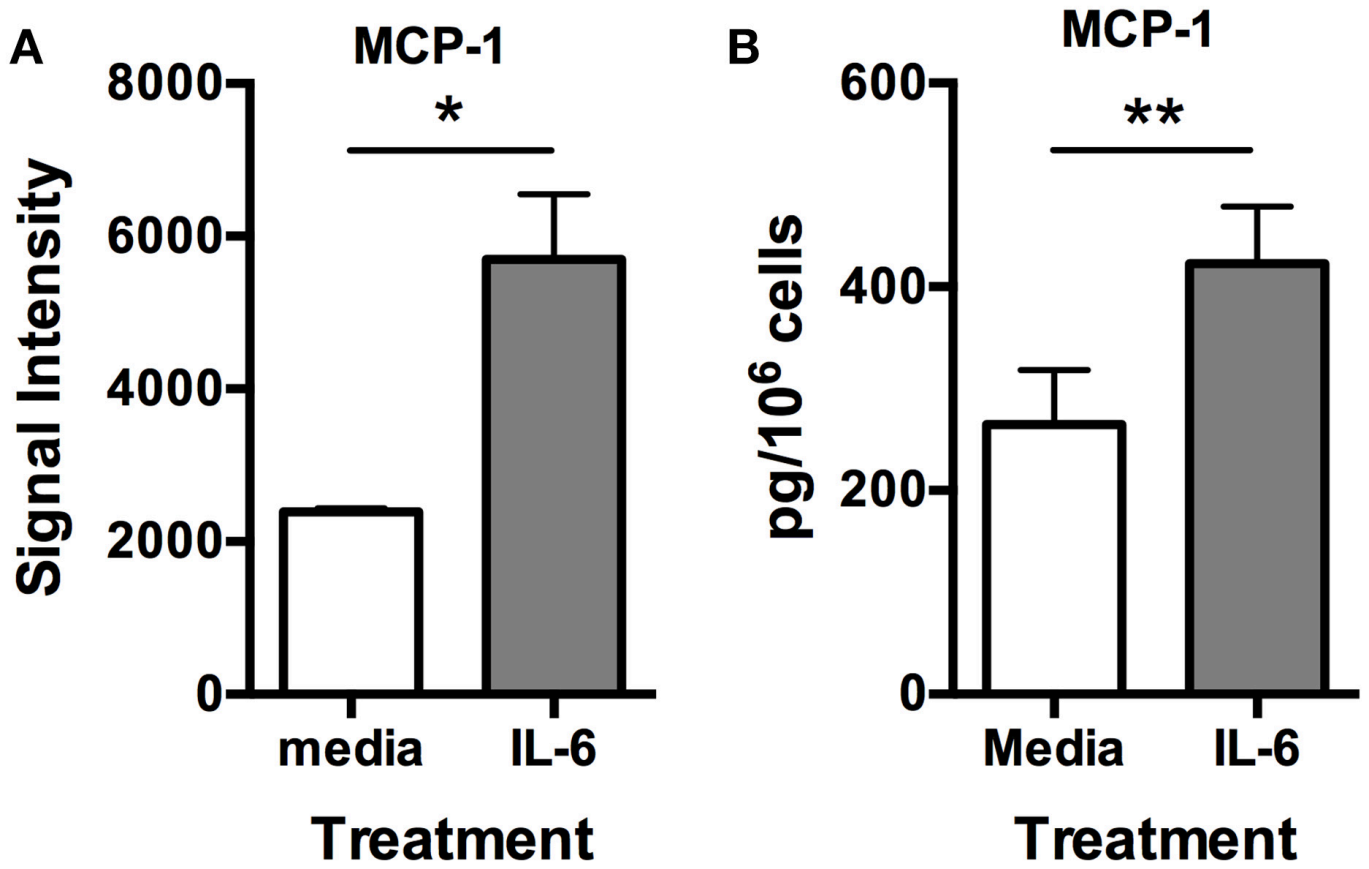
IL-6 induces MCP-1 secretion from human skin mast cells. Human skin mast cells were treated or not with IL-6 (100 ng/ml) for 24 h, and the media was analyzed with the Human Angiogenesis Proteome Profiler™ Array (R&D Systems) **(A)**, or treated for 6 h, and the media analyzed with ELISA for MCP-1 **(B)**. The data sets in **(A)** represent the mean signal intensity ± S.E.M. (*n* = 4) obtained from membrane arrays incubated with media from individual mast cell cultures prepared from skin tissue of different donors. The graph bars in **(B)** represent MCP-1 concentration ± S.E.M. (*n* = 3) obtained from individual mast cell cultures isolated from skin tissue of different donors. Significance was determined with Student's *t*-test. ^*^p < 0.05; ^**^p < 0.01.

Previously, we demonstrated that IL-6 could induce VEGF synthesis and secretion from human skin mast cells (18). Therefore, to determine if angiogenesis-related factors other than VEGF were also induced with IL-6, we cultured human skin-derived mast cells without and with IL-6 (100 ng/ml) for 24 h, and analyzed the cell-free medium with the Human Angiogenesis Proteome Profiler™ Array. Interestingly, among all the proteins detectable with the array, the most noteworthy observation was a robust and significant increase in MCP-1 in the presence of added IL-6 (Figure S2 and Figure 5A). To validate the array data, human skin-derived mast cells were cultured without and with IL-6 (100 ng/ml) for 6 h, and MCP-1 in the cell-free medium was measured with ELISA. As shown in Figure 5B, mast cells cultured with MCP-1 secreted significantly more MCP-1 compared to those cultured without IL-6. These findings demonstrate a previously unknown ability of IL-6 to potentiate MCP-1 secretion from human skin-derived mast cells.

## Discussion

Mast cells are classically known to be involved in allergic and inflammatory reactions. However, their ability to secrete VEGF, and to localize in and around solid tumors has suggested a role for mast cells in tumorigenesis. Here, we report the novel observation that human skin-derived mast cells spontaneously secrete several proteins that, in addition to VEGF, have the potential to influence angiogenesis and vascular development. Specifically, we identified CXCL16, DPPIV, Endothelin-1, GM-CSF, IL-8, MCP-1, Pentraxin 3, Serpin E1, Serpin F1, TIMP-1, Thrombospondin-1, and uPA as being spontaneously secreted at very high levels from human skin-derived mast cells. Functionally, CXCL16 (34), Endothelin-1 (35, 36), GM-CSF (37), IL-8 (38), MCP-1 (39), and uPA (40) are reportedly pro-angiogenic, whereas Serpin F1 (Pigment Epithelium-Derived Factor) and Thrombospondin-1 are considered endogenous inhibitors of angiogenesis (41). However, some studies indicate a pro-angiogenic property of Thrombospondin-1 (42). Similarly, Pentraxin-3 (43), DPPIV (44–46), TIMP-1 (36, 47), and Serpin E1 (Plasminogen Activator Inhibitor) (48, 49) have each also been implicated as having both pro- and anti-angiogenesis properties. Additional studies will determine the specific contribution of each of these mast cell-derived proteins in tumor development, but the fact that most of these factors were secreted at levels much greater than VEGF suggests that they could potentially have a greater influence on mast cell-mediated angiogenesis and tumor development than VEGF.

Our finding that Endothelin-1, GM-CSF, IL-8, MCP-1, and VEGF secretion was almost completely abolished in the absence of SCF, and that Pentraxin 3, Serpin E1, Serpin F1, TIMP-1, and Thrombospondin-1 secretion was also diminished, albeit to a much lower extent, indicates that c-kit signals play a significant role in the secretion of these factors. On the other hand, it is possible that the observed inhibition in secretion of some proteins in the absence of SCF is simply a reflection of an overall reduction in cellular function. However, this appears to be unlikely since not all proteins were equally affected with some proteins being markedly more sensitive to SCF withdrawal than others. The fact that CXCL16, DPPIV, and uPa secretion was completely unaffected by SCF withdrawal indicates that loss of kit signaling rather than a global reduction in cellular function is a more likely explanation for the unequal reduction in protein secretion. Interestingly, the fact that mast cells require SCF for survival suggests that the existence of mast cells and secretion of these angiogenesis regulating factors go hand-in-hand, perhaps indicating a primordial role for mast cells in angiogenesis and neovascularization in development.

FcεRI signals are generally considered positive signals that result in the induction of cytokine gene expression and protein production, and other functional events. Therefore, it was surprising to find that secretion of CXCL16, Endothelin-1, Serpin F1, Thrombospondin-1, MCP-1 and Pentraxin-1 was significantly inhibited following FcεRI crosslinking. We ruled out the possibility that this was due to interference by unbound NP-BSA with the ELISA assay. However, another possibility is that the observed reduction was due to cleavage by a protease not susceptible to inhibition with SBTI that are released from mast cells following activation. If so, the fact that FcεRI crosslinking led to increased secretion of GM-CSF, IL-8, Serpin E1, and VEGF production, and also induced the secretion of Amphiregulin and MMP-8 indicates that not all proteins were susceptible to degradation by the putative protease. A more intriguing possibility is that FcεRI signals act to inhibit the spontaneous secretion of these proteins, perhaps by interfering with kit signaling or other unknown signals. It is interesting to speculate on whether signals from FcεRI, or other receptors, serve to negatively regulate the intrinsic spontaneous secretion of angiogenesis regulating proteins from mast cells. The current study also demonstrated that treatment with IL-6 significantly induced the secretion of pro-angiogenic MCP-1 from human skin-derived mast cells. We previously demonstrated that IL-6 could induce the production of VEGF (18), which we also observed here. Together, these data together indicate that IL-6 can indirectly induce mast cell-mediated angiogenesis by inducing the secretion of MCP-1 and VEGF.

Mast cells are heterogeneous population of cells (50). In humans, two subpopulations were identified over 30 years ago based on their expression of chymase (C) and tryptase (T) in their cytoplasmic granules: MC_TC_ and MC_T_ (51). Immunohistochemical analysis demonstrated that the MC_TC_ type expresses both tryptase and chymase whereas the MC_T_ type expresses only tryptase. MC_TC_ type cells are the predominant type in the skin whereas MC_T_ cells make up the majority of mast cells in lung and mucosa of the small intestine. Ultrastructural analysis by immunoelectron microscopy revealed that both MC_T_ and MC_TC_ mast cells have large numbers of cytoplasmic granules. However, MC_TC_ mast cell granules are more uniformly electron dense and larger than MC_T_ granules, which are more variable in shape than MC_TC_ granules (52).

Cultured skin-derived mast cells, such as those used in this study, have been extensively studied and characterized, and are believed to be representative of *in situ*-matured MC_TC_ mast cells. In addition to expressing both chymase and tryptase, various studies have demonstrated that cultured skin-derived mast cells express carboxypeptidase and complement factor 5a receptor (CD88) whereas lung MC_T_ cells do not (53, 54), A3AR adenosine receptor at significantly lower levels compared with dispersed lung mast cells (55), complement factors C3a and C5a (56), FcγRIIa (57), gp130, and membrane-bound IL-6Rα (18). However, whether skin-derived mast cells that have been cultured for an extensive period of time are phenotypically identical to those that were initially dispersed from the skin tissue or resident in skin tissue is not clear. It is also possible that cultured skin-derived mast cells developed *in vitro* from progenitors and are distinct from *in situ*-matured mast cells that were originally isolated. Indeed, mast cell progenitors have been found in human skin follicles (58). Therefore, longstanding question of whether cultured skin-derived mast cells are phenotypically and functionally identical to tissue resident mast cells remains to be answered.

Overall, this study shows that human skin-derived mast cells have an intrinsic ability to spontaneously secrete several proteins, in addition to VEGF, with the potential to regulate blood vessel formation. The study further suggests that extracellular signals from FcεRI and very likely other receptors expressed on the mast cell surface could serve to modulate the spontaneous secretion of some proteins. In addition to supporting the known role for mast cell in tumorigenesis, these findings raise the possibility of a critical role for mast cells in development.

## Data Availability

All datasets generated for this study are included in the manuscript and/or the Supplementary Files.

## Ethics Statement

This study has been reviewed by the Institutional Review Board (IRB) of the University of South Carolina, and has been deemed exempt from Protection of Human Subjects Research regulations. Therefore, it does not qualify as human subjects research. The skin tissues used to obtain the mast cells for this study were purchased from the NCI-sponsored Cooperative Human Tissue Network (CHTN). Subjects from whom tissues are obtained by the CHTN are consented by that organization. Subject identifiers are not provided to the investigators.

## Author Contributions

CM performed the experiments and assisted with data analysis. ZM assisted with experiments and immunoassay development. CM and ZM isolated and purified the mast cells. GG directed the project, analyzed the data, and wrote the manuscript.

### Conflict of Interest Statement

The authors declare that the research was conducted in the absence of any commercial or financial relationships that could be construed as a potential conflict of interest.

## References

[1] Kirshenbaum AS Goff JP Semere T Foster B Scott LM Metcalfe DD. Demonstration that human mast cells arise from a progenitor cell population that is CD34(+), c-kit(+), and expresses aminopeptidase N (CD13). Blood. (1999) 94:2333–42. 10498605

[2] Galli SJ Tsai M. IgE and mast cells in allergic disease. Nat Med. (2012) 18:693–704. 10.1038/nm.2755 22561833 PMC3597223

[3] Varricchi G Galdiero MR Loffredo S Marone G Iannone R Marone G. Are mast cells MASTers in cancer? Front Immunol. (2017) 8:424. 10.3389/fimmu.2017.00424 28446910 PMC5388770

[4] Albini A Bruno A Noonan DM Mortara L. Contribution to tumor angiogenesis from innate immune cells within the tumor microenvironment: implications for immunotherapy. Front Immunol. (2018) 9:527. 10.3389/fimmu.2018.00527 29675018 PMC5895776

[5] Melillo RM Guarino V Avilla E Galdiero MR Liotti F Prevete N. Mast cells have a protumorigenic role in human thyroid cancer. Oncogene. (2010) 29:6203–15. 10.1038/onc.2010.348 20729915

[6] Visciano C Liotti F Prevete N Cali G Franco R Collina F. Mast cells induce epithelial-to-mesenchymal transition and stem cell features in human thyroid cancer cells through an IL-8-Akt-Slug pathway. Oncogene. (2015) 34:5175–86. 10.1038/onc.2014.441 25619830

[7] Yano H Kinuta M Tateishi H Nakano Y Matsui S Monden T. Mast cell infiltration around gastric cancer cells correlates with tumor angiogenesis and metastasis. Gastric Cancer. (1999) 2:26–32. 11957067 10.1007/s101200050017

[8] Kondo K Muramatsu M Okamoto Y Jin D Takai S Tanigawa N. Expression of chymase-positive cells in gastric cancer and its correlation with the angiogenesis. J Surg Oncol. (2006) 93:36–42; discussion 42–3. 10.1002/jso.20394 16353179

[9] Ammendola M Sacco R Donato G Zuccalà V Russo E Luposella M. Mast cell positivity to tryptase correlates with metastatic lymph nodes in gastrointestinal cancer patients treated surgically. Oncology. (2013) 85:111–6. 10.1159/000351145 23887206

[10] Rao Q Chen Y Yeh C-R Ding J Li L Chang C. Recruited mast cells in the tumor microenvironment enhance bladder cancer metastasis via modulation of ERβ/CCL2/CCR2 EMT/MMP9 signals. Oncotarget. (2016) 7:7842–55. 10.18632/oncotarget.5467 26556868 PMC4884958

[11] Dabiri S Huntsman D Makretsov N Cheang M Gilks B Bajdik C. The presence of stromal mast cells identifies a subset of invasive breast cancers with a favorable prognosis. Mod Pathol. (2004) 17:690–5. 10.1038/modpathol.3800094 15044916

[12] Amini R-M Aaltonen K Nevanlinna H Carvalho R Salonen L Heikkilä P. Mast cells and eosinophils in invasive breast carcinoma. BMC Cancer. (2007) 7:165. 10.1186/1471-2407-7-165 17727696 PMC2048965

[13] Rajput AB Turbin DA Cheang MC Voduc DK Leung S Gelmon KA. Stromal mast cells in invasive breast cancer are a marker of favourable prognosis: a study of 4,444 cases. Breast Cancer Res Treat. (2008) 107:249–57. 10.1007/s10549-007-9546-3 17431762 PMC2137942

[14] Mukai K Tsai M Saito H Galli SJ. Mast cells as sources of cytokines, chemokines, and growth factors. Immunol Rev. (2018) 282:121–50. 10.1111/imr.12634 29431212 PMC5813811

[15] Marone G Varricchi G Loffredo S Granata F. Mast cells and basophils in inflammatory and tumor angiogenesis and lymphangiogenesis. Eur J Pharmacol. (2016) 778:146–51. 10.1016/j.ejphar.2015.03.088 25941082

[16] Grützkau A Krüger-Krasagakes S Baumeister H Schwarz C Kögel H Welker P. Synthesis, storage, and release of vascular endothelial growth factor/vascular permeability factor (VEGF/VPF) by human mast cells: implications for the biological significance of VEGF206. Mol Biol Cell. (1998) 9:875–84. 9529385 10.1091/mbc.9.4.875 PMC25314

[17] Boesiger J Tsai M Maurer M Yamaguchi M Brown LF Claffey KP. Mast cells can secrete vascular permeability factor/vascular endothelial cell growth factor and exhibit enhanced release after immunoglobulin E-dependent upregulation of fc epsilon receptor I expression. J Exp Med. (1998) 188:1135–45. 9743532 10.1084/jem.188.6.1135 PMC2212544

[18] McHale C Mohammed Z Deppen J Gomez G. Interleukin-6 potentiates FcεRI-induced PGD2biosynthesis and induces VEGF from human *in situ*-matured skin mast cells. Biochim Biophys Acta. (2018) 1862:1069–78. 10.1016/j.bbagen.2018.01.020 29410184 PMC5866211

[19] Detoraki A Staiano RI Granata F Giannattasio G Prevete N de Paulis A. Vascular endothelial growth factors synthesized by human lung mast cells exert angiogenic effects. J Allergy Clin Immunol. (2009) 123:1142–9, 1149.e1–5. 10.1016/j.jaci.2009.01.044 19275959

[20] Abdel-Majid RM Marshall JS. Prostaglandin E2 induces degranulation-independent production of vascular endothelial growth factor by human mast cells. J Immunol. (2004) 172:1227–36. 10.4049/jimmunol.172.2.1227 14707101

[21] Nakayama T Yao L Tosato G. Mast cell-derived angiopoietin-1 plays a critical role in the growth of plasma cell tumors. J Clin Invest. (2004) 114:1317–25. 10.1172/JCI22089 15520864 PMC524229

[22] Sismanopoulos N Delivanis DA Alysandratos KD Angelidou A Vasiadi M Therianou A. IL-9 induces VEGF secretion from human mast cells and IL-9/IL-9 receptor genes are overexpressed in atopic dermatitis. PLoS ONE. (2012) 7:e33271. 10.1371/journal.pone.0033271 22413008 PMC3297631

[23] Cao J Papadopoulou N Kempuraj D Boucher WS Sugimoto K Cetrulo CL. Human mast cells express corticotropin-releasing hormone (CRH) receptors and CRH leads to selective secretion of vascular endothelial growth factor. J Immunol. (2005) 174:7665–75. 10.4049/jimmunol.174.12.7665 15944267

[24] Feoktistov I Ryzhov S Goldstein AE Biaggioni I. Mast cell-mediated stimulation of angiogenesis: cooperative interaction between A2B and A3 adenosine receptors. Circ Res. (2003) 92:485–92. 10.1161/01.RES.0000061572.10929.2D 12600879

[25] Ribatti D. Mast cells as therapeutic target in cancer. Eur J Pharmacol. (2016) 778:152–7. 10.1016/j.ejphar.2015.02.056 25917325

[26] Troupin A Shirley D Londono-Renteria B Watson AM McHale C Hall A. A role for human skin mast cells in dengue virus infection and systemic spread. J Immunol. (2016) 197:4382–91. 10.4049/jimmunol.1600846 27799312

[27] Kirshenbaum AS Goff JP Kessler SW Mican JM Zsebo KM Metcalfe DD. Effect of IL-3 and stem cell factor on the appearance of human basophils and mast cells from CD34^+^ pluripotent progenitor cells. J Immunol. (1992) 148:772–7. 1370517

[28] Valent P Spanblöchl E Sperr WR Sillaber C Zsebo KM Agis H. Induction of differentiation of human mast cells from bone marrow and peripheral blood mononuclear cells by recombinant human stem cell factor/kit-ligand in long-term culture. Blood. (1992) 80:2237–45. 1384799

[29] Irani AM Nilsson G Miettinen U Craig SS Ashman LK Ishizaka T. Recombinant human stem cell factor stimulates differentiation of mast cells from dispersed human fetal liver cells. Blood. (1992) 80:3009–21. 1281684

[30] Mitsui H Furitsu T Dvorak AM Irani AM Schwartz LB Inagaki N. Development of human mast cells from umbilical cord blood cells by recombinant human and murine c-kit ligand. Proc Natl Acad Sci USA. (1993) 90:735–9. 7678463 10.1073/pnas.90.2.735 PMC45740

[31] Durand B Migliaccio G Yee NS Eddleman K Huima-Byron T Migliaccio AR. Long-term generation of human mast cells in serum-free cultures of CD34^+^ cord blood cells stimulated with stem cell factor and interleukin-3. Blood. (1994) 84:3667–74. 7524746

[32] Zhao W Oskeritzian CA Pozez AL Schwartz LB. Cytokine production by skin-derived mast cells: endogenous proteases are responsible for degradation of cytokines. J Immunol. (2005) 175:2635–42. 10.4049/jimmunol.175.4.2635 16081839

[33] Oskeritzian CA Zhao W Pozez AL Cohen NM Grimes M Schwartz LB Neutralizing endogenous IL-6 renders mast cells of the MCT type from lung, but not the MCTC type from skin and lung, susceptible to human recombinant IL-4-induced apoptosis. J Immunol. (2004) 172:593–600. 10.4049/jimmunol.172.1.593 14688371

[34] Yu X Zhao R Lin S Bai X Zhang L Yuan S. CXCL16 induces angiogenesis in autocrine signaling pathway involving hypoxia-inducible factor 1α in human umbilical vein endothelial cells. Oncol Rep. (2016) 35:1557–65. 10.3892/or.2015.4520 26707275

[35] Lankhorst S Danser AHJ van den Meiracker AH. Endothelin-1 and antiangiogenesis. Am J Physiol Regul Integr Comp Physiol. (2016) 310:R230–4. 10.1152/ajpregu.00373.2015 26511523

[36] Cabral T Mello LGM Lima LH Polido J Regatieri CV Belfort R. Retinal and choroidal angiogenesis: a review of new targets. Int J Retina Vitreous. (2017) 3:31. 10.1186/s40942-017-0084-9 28835854 PMC5563895

[37] Ribatti D Tamma R. Hematopoietic growth factors and tumor angiogenesis. Cancer Lett. (2019) 440–441:47–53. 10.1016/j.canlet.2018.10.008 30312730

[38] Liu Q Li A Tian Y Wu JD Liu Y Li T. The CXCL8-CXCR1/2 pathways in cancer. Cytokine Growth Factor Rev. (2016) 31:61–71. 10.1016/j.cytogfr.2016.08.002 27578214 PMC6142815

[39] Adini I Adini A Bazinet L Watnick RS Bielenberg DR D'Amato RJ. Melanocyte pigmentation inversely correlates with MCP-1 production and angiogenesis-inducing potential. FASEB J. (2015) 29:662–70. 10.1096/fj.14-255398 25406462 PMC4314224

[40] Stepanova V Jayaraman P-S Zaitsev SV Lebedeva T Bdeir K Kershaw R. Urokinase-type plasminogen activator (uPA) promotes angiogenesis by attenuating proline-rich homeodomain protein (PRH) transcription factor activity and de-repressing vascular endothelial growth factor (VEGF) receptor expression. J Biol Chem. (2016) 291:15029–45. 10.1074/jbc.M115.678490 27151212 PMC4946921

[41] Farnoodian M Wang S Dietz J Nickells RW Sorenson CM Sheibani N. Negative regulators of angiogenesis: important targets for treatment of exudative AMD. Clin Sci. (2017) 131:1763–80. 10.1042/CS20170066 28679845 PMC6016847

[42] Byrne GJ Hayden KE McDowell G Lang H Kirwan CC Tetlow L. Angiogenic characteristics of circulating and tumoural thrombospondin-1 in breast cancer. Int J Oncol. (2007) 31:1127–32. 10.3892/ijo.31.5.1127 17912439

[43] Presta M Foglio E Churruca Schuind A Ronca R. Long pentraxin-3 modulates the angiogenic activity of fibroblast growth factor-2. Front Immunol. (2018) 9:2327. 10.3389/fimmu.2018.02327 30349543 PMC6187966

[44] Lei Y Hu L Yang G Piao L Jin M Cheng X. Dipeptidyl peptidase-IV inhibition for the treatment of cardiovascular disease–recent insights focusing on angiogenesis and neovascularization. Circ J. (2017) 81:770–6. 10.1253/circj.CJ-16-1326 28344207

[45] Whittam AJ Maan ZN Duscher D Barrera JA Hu MS Fischer LH. Small molecule inhibition of dipeptidyl peptidase-4 enhances bone marrow progenitor cell function and angiogenesis in diabetic wounds. Transl Res. (2019) 205:51–63. 10.1016/j.trsl.2018.10.006 30452888 PMC7252504

[46] Qin C-J Zhao L-H Zhou X Zhang H-L Wen W Tang L. Inhibition of dipeptidyl peptidase IV prevents high fat diet-induced liver cancer angiogenesis by downregulating chemokine ligand 2. Cancer Lett. (2018) 420:26–37. 10.1016/j.canlet.2018.01.064 29409972

[47] Rojiani MV Ghoshal-Gupta S Kutiyanawalla A Mathur S Rojiani AM. TIMP-1 overexpression in lung carcinoma enhances tumor kinetics and angiogenesis in brain metastasis. J Neuropathol Exp Neurol. (2015) 74:293–304. 10.1097/NEN.0000000000000175 25756591

[48] Bhakuni T Ali MF Ahmad I Bano S Ansari S Jairajpuri MA. Role of heparin and non heparin binding serpins in coagulation and angiogenesis: a complex interplay. Arch Biochem Biophys. (2016) 604:128–42. 10.1016/j.abb.2016.06.018 27372899

[49] Devy L Blacher S Grignet-Debrus C Bajou K Masson V Gerard RD. The pro- or antiangiogenic effect of plasminogen activator inhibitor 1 is dose dependent. FASEB J. (2002) 16:147–54. 10.1096/fj.01-0552com 11818362

[50] Lawrence ID Warner JA Cohan VL Hubbard WC Kagey-Sobotka A Lichtenstein LM. Purification and characterization of human skin mast cells. Evidence for human mast cell heterogeneity. J Immunol. (1987) 139:3062–9. 2444649

[51] Irani AA Schechter NM Craig SS DeBlois G Schwartz LB. Two types of human mast cells that have distinct neutral protease compositions. Proc Natl Acad Sci USA. (1986) 83:4464–8. 3520574 10.1073/pnas.83.12.4464 PMC323754

[52] Craig SS Schechter NM Schwartz LB. Ultrastructural analysis of human T and TC mast cells identified by immunoelectron microscopy. Lab Invest. (1988) 58:682–91. 2454349

[53] Irani AM Goldstein SM Wintroub BU Bradford T Schwartz LB. Human mast cell carboxypeptidase. Selective localization to MCTC cells. J Immunol. (1991) 147:247–53. 2051021

[54] Oskeritzian CA Zhao W Min H-K Xia H-Z Pozez A Kiev J. Surface CD88 functionally distinguishes the MCTC from the MCT type of human lung mast cell. J Allergy Clin Immunol. (2005) 115:1162–8. 10.1016/j.jaci.2005.02.022 15940129 PMC1460014

[55] Gomez G Zhao W Schwartz LB. Disparity in FcεRI-induced degranulation of primary human lung and skin mast cells exposed to adenosine. J Clin Immunol. (2011) 31:479–87. 10.1007/s10875-011-9517-7 21437670 PMC3417298

[56] Fukuoka Y Hite MR Dellinger AL Schwartz LB. Human skin mast cells express complement factors C3 and C5. J Immunol. (2013) 191:1827–34. 10.4049/jimmunol.1202889 23833239

[57] Zhao W Kepley CL Morel PA Okumoto LM Fukuoka Y Schwartz LB Fc gamma RIIa, not Fc gamma RIIb, is constitutively and functionally expressed on skin-derived human mast cells. J Immunol. (2006) 177:694–701. 10.4049/jimmunol.177.1.694 16785568 PMC2176083

[58] Kumamoto T Shalhevet D Matsue H Mummert ME Ward BR Jester JV. Hair follicles serve as local reservoirs of skin mast cell precursors. Blood. (2003) 102:1654–60. 10.1182/blood-2003-02-0449 12738661

