# Supplementary material for: Human Skin-Derived Mast Cells Spontaneously Secrete Several Angiogenesis-Related Factors
Source: Front Immunol. 2019 Jun 25;10:1445. doi: 10.3389/fimmu.2019.01445 (PMC6603178; doi:10.3389/fimmu.2019.01445)
Supplement: Supplementary file 1 [file Data_Sheet_1.docx]

***Supplementary Material***


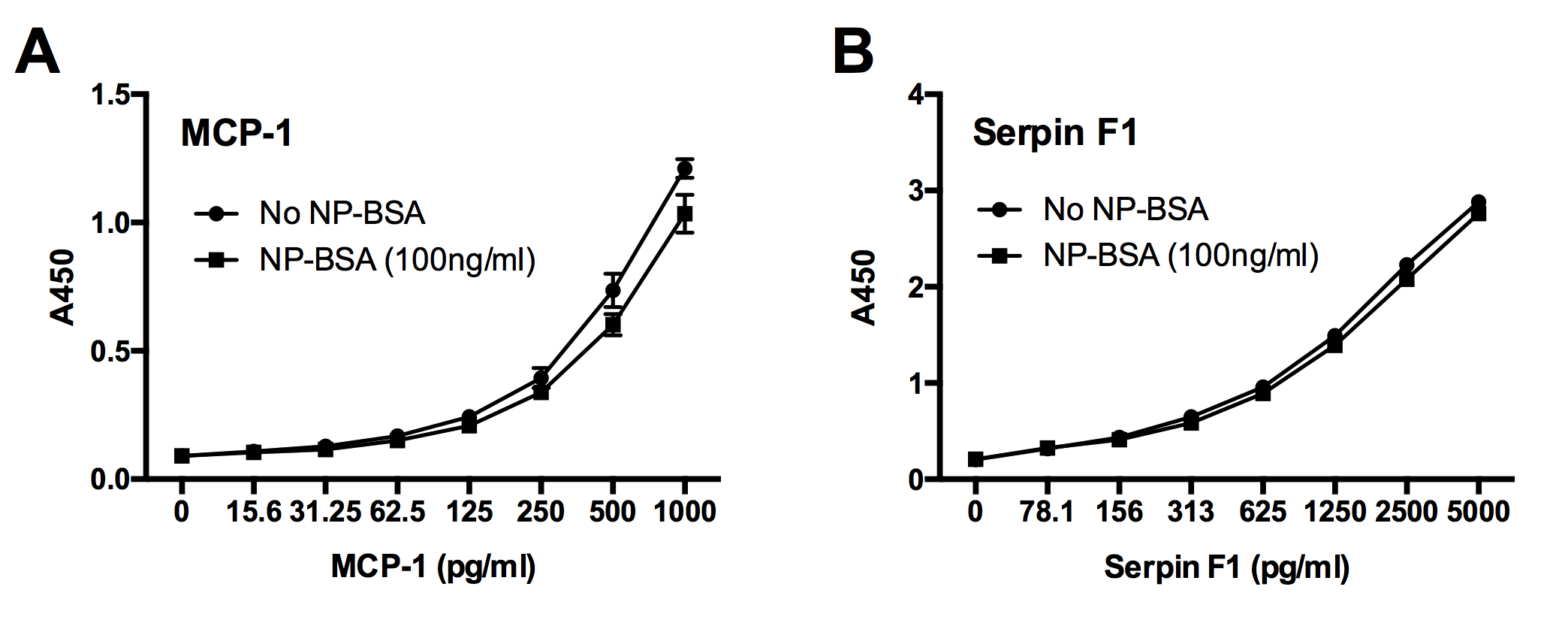


**Figure S1. Effect of free NP-BSA on MCP-1 and Serpin F1 ELISA.** To determine if the observed inhibition in MCP-1 and Serpin F1 following FcεRI stimulation was due to interference by non-bound NP-BSA with the ELISA assay, standard curves were prepared with known concentrations of MCP-1 (A, n=2) and Serpin F1 (B, n=1) without and with NP-BSA (100 ng/ml). The assay was developed according to the manufacturers' instructions.


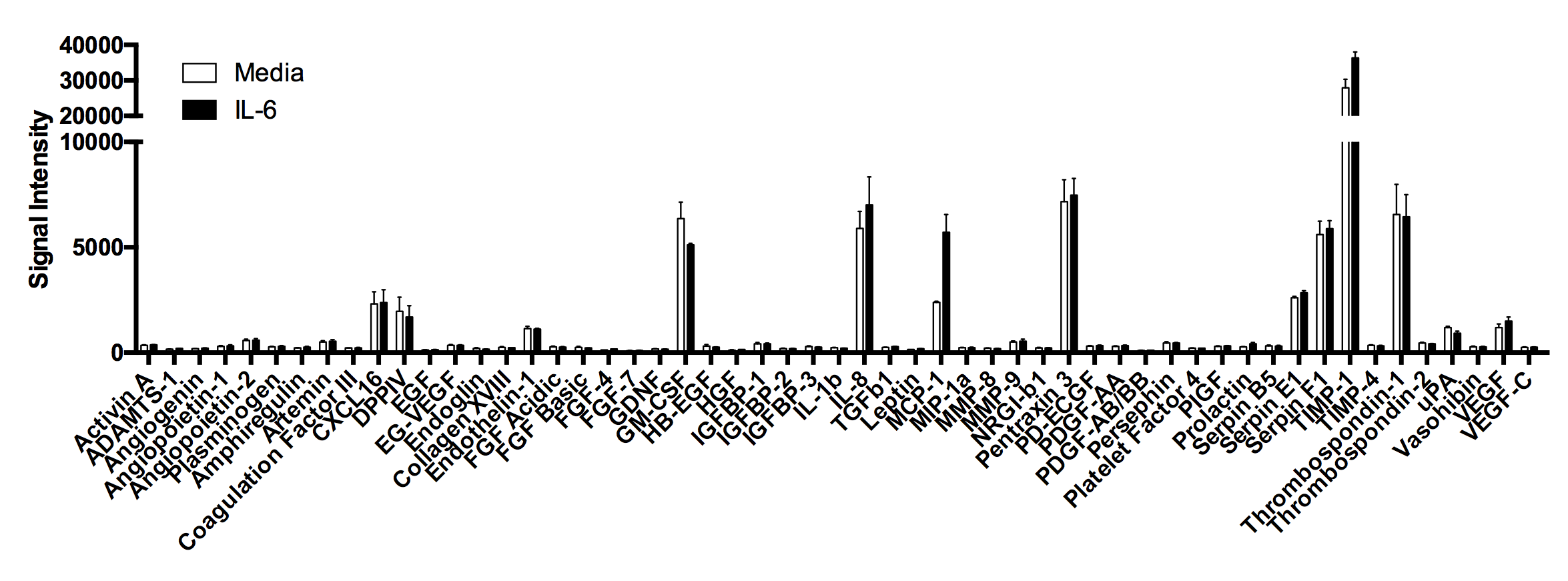


**Figure S2. Effect of IL-6 on secretion of angiogenesis-related factors.** Human skin mast cells were treated, or not, with IL-6 (100 ng/ml) for 24 h, and the collected cell-free media was analyzed with the Human Angiogenesis Proteome Profiler™ Array (R&D Systems). Graph bars represent data from 4 different arrays probed with media from individual mast cell cultures prepared from two different donors.
